# Variability in Seed Traits in a Collection of *Cannabis sativa* L. Genotypes

**DOI:** 10.3389/fpls.2016.00688

**Published:** 2016-05-20

**Authors:** Incoronata Galasso, Roberto Russo, Sergio Mapelli, Elena Ponzoni, Ida M. Brambilla, Giovanna Battelli, Remo Reggiani

**Affiliations:** ^1^Institute of Agricultural Biology and Biotechnology, CNRMilano, Italy; ^2^Institute of Science of Food Production, CNRMilano, Italy

**Keywords:** protein, antinutritional compounds, fatty acids, tocopherols, polyphenols, antioxidant activity, genotype diversity

## Abstract

The seed of *Cannabis sativa* L. is an expanding source of proteins and oil for both humans and animals. In this study, the proximate composition of a collection of hemp cultivars and accessions of different geographical origins grown under the same conditions for 1 year was analyzed in order to identify potential accessions to improve hemp cultivars. Fatty acids, tocopherols, and antinutritional components, as well as concentrations of crude protein and oil were quantified. The seed oil concentrations varied between 285 and 360 g kg^−1^ dry seed (DS), while crude protein ranged between 316 and 356 g kg^−1^ dry matter (DM). The seed oil was mainly composed of unsaturated fatty acids and, as expected, the dominant fatty acids were linoleic and α-linolenic acid. A high variability among the cultivars and accessions was also detected for polyphenolic content which ranged from 5.88 to 10.63 g kg^−1^ DM, cv. Felina was the richest, whereas cv. Finola had the lowest polyphenolic content. Regarding antinutritional compounds in seed, a high variability was detected among all genotypes analyzed and phytic acid was particularly abundant (ranging between 43 and 75 g kg^−1^ DM). In conclusion, our results reveal noticeable differences among hemp seed genotypes for antinutritional components, oil and protein content. Collectively, this study suggests that the hemp seed is an interesting product in terms of protein, oil and antioxidant molecules but a reduction of phytic acid would be desirable for both humans and monogastric animals. The high variability detected among the different genotypes indicates that an improvement of hemp seed might be possible by conventional and/or molecular breeding.

## Introduction

*Cannabis sativa* L. (hemp) is a wind-pollinated annual plant that originated in central Asia (Li, 1973, 1974; Mukherjee et al., 2008). Hemp, naturally, is a dioecious crop, but some monoecious cultivars have been obtained as a result of earlier breeding efforts. Hemp is an ancient crop that has been cultivated worldwide until the early twentieth century, after which its cultivation declined. Recently, interest in this multipurpose crop delivering fibers, shives, and seeds, has been renewed by an increasing demand not only for natural fibers but also for the high content and quality of seed protein and oil. Hemp seed contains 25–35% oil, 20–25% protein, 20–30% carbohydrates, 10–15% insoluble fiber, vitamins, and minerals such as phosphorus, potassium, magnesium, sulfur, and calcium (Deferne and Pate, 1996; Callaway, 2004; House et al., 2010). The increasing demand for vegetable oils and proteins, along with current awareness about their nutritional and functional role in human diet, has made indispensable to characterize new plant sources. In this regard, hemp seed contains all the essential amino acids and fatty acids necessary to maintain healthy human life, and it might be a new good source of nutrients for both humans and livestock (Osburn, 1992; Deferne and Pate, 1996; Callaway, 2004; Russo and Reggiani, 2015a).

The principal value of hemp seed oil resides in its fatty acid composition. It contains the two dietary essential fatty acids: linoleic acid (LA; 18:2ω6) and the α-linolenic acid (ALA; 18:3ω3) in the ratio of 2.5–3:1, which has been claimed as ideal for human nutrition (Simopoulos, 2008). In addition, the biological metabolites of LA and ALA, the γ-linolenic acid (GLA; 18:3ω6) and stearidonic acid (SDA; 18:4ω3) are also present in hemp seed oil, which makes the nutritional value of hemp seed superior to other seed oils (Leizer et al., 2000). Both GLA and SDA act as precursors for the rapid synthesis of longer chain fatty acids which are involved in many important biological processes (Guil-Guerrero et al., 2010).

Furthermore, hemp seeds are also an excellent nutritional source of high quality proteins (Russo and Reggiani, 2015a), as they are easily digested, absorbed, and utilized (House et al., 2010). The two main proteins in hemp seeds are the globulin edestin and albumin (Callaway, 2004; Tang et al., 2006). Edestin, which accounts for about 60–80% of the total protein content (Odani and Odani, 1998; Tang et al., 2006), has been the subject of intensive investigation for a long time. Recently, an accurate characterization of edestin at biochemical and molecular level has been done by Kim and Lee (2011) and Docimo et al. (2014). This storage protein, which contains exceptionally high levels of arginine and glutamic acids (Russo and Reggiani, 2015a), is easily digested and rich in all essential amino acids (Callaway, 2004; House et al., 2010; Docimo et al., 2014).

However, the nutritional quality of plant proteins, as measured by their amino acid composition and digestibility, is influenced by numerous factors. The amino acid composition may be influenced by genotypic variability or agronomic conditions such as soil fertility and postharvest processing that alters the ratio of seed components (e.g., shelling). The digestibility of proteins may be affected by protein structure, presence of antinutritional compounds and high temperature processing (Sarwar, 1997). Recently, it has been demonstrated that hemp meal (a by-product obtained from mechanical or solvent extraction of the seed oil) contains some antinutritional compounds that need to be considered carefully in feeding applications (Russo and Reggiani, 2013). In hemp seed, the content of phytic acid (inositol hexaphosphate, IP_6_) and trypsin inhibitors appeared fairly high compared to a number of other antinutritional compounds analyzed (Russo and Reggiani, 2015b). Phytic acid is the main organic form of phosphorus present in plant seeds. Its presence reduces protein digestibility and increases the excretion of endogenous nitrogen, amino acids and minerals, in particular bivalent cations (Cowieson et al., 2004). The trypsin inhibitors are present in many species of *Leguminosae, Brassicaceae,* and *Gramineae*, and reduce the biological activity of trypsin enzyme which is involved in the breakdown of many different proteins (Guillamón et al., 2008).

Therefore, in this work several hemp cultivars and accessions of different origins, grow in the same environment for 1 year, have been characterized for quantity and quality of seed oil, protein content, antinutritional compounds, polyphenols, tocopherols, and antioxidant activity. The objective of this study was the evaluation of these biochemical traits in order to identify potential accessions to improve hemp cultivars. To achieve this goal, we analyzed the seeds of 20 genotypes composed by the most used non-drug hemp cultivars that are allowed to be cultivated in Europe and a collection of hemp accessions obtained from genebanks.

## Materials and methods

### Plant material

Seeds of hemp cultivars (cvs) and accessions were kindly provided by the genebank of IPK Gatersleben (Leibniz Institute of Plant Genetics and Crop Plant Research) (http://gbis.ipk-gatersleben.de/), the Centro di Ricerca per le Colture Industriali (CRA-CIN, Italy) and by seed companies (Table 1). All genotypes were sown at the beginning of spring (30 March 2014) in pots of about 1 m^2^ with three replications. The pots were fertilized with 80 kg ha^−1^ N after the seedlings had emerged. The average air temperature was 19.4°C (30 March–31 October 2014). During summer the plants were irrigated when necessary. In order to avoid cross-pollination among the different cvs and accessions, all pots were covered with two layers of cloth (~ 200 mesh; Virexgomma, Somma Lombardo, Varese, Italy) during flowering time. Seeds were harvested at maturity from August to October. The harvesting date depended on the genotype. After harvesting, seeds were forced-air-dried (30°C) in a ventilated chamber until 8% seed moisture was reached. Thousand seed weight (TSW) was established on five replicates of 100 seeds each. Seeds were manually counted and weighed. During the flowering stage, a pool of inflorescences (flowers and leaves) were collected from each pot. The Δ^9^-tetrahydrocannabinol (THC) concentration was measured in each genotype by the HPTLC densitometry method according to Fischedick et al. (2009).

**Table 1:** **List of hemp accessions (from CAN19 to CAN58) and cultivars (from cv. Finola to cv. Kc Dora) analyzed in this study**.

**Hemp genotypes**	**Sex type**	**Origin**	**Flowering time**	**Supply source**
*C. sativa* CAN19	D^*^	Italy	End July	IPK^a^
*C. sativa* CAN20	D	Korea	End July	IPK
*C. sativa* CAN24	D	Italy	End July	IPK
*C. sativa* CAN26	D	Turkey	End July	IPK
*C. sativa* CAN39	D	China	End July	IPK
*C. sativa* CAN40	D	Italy	End July	IPK
*C. sativa* CAN48	D	Italy	End July	IPK
*C. sativa* CAN51	D	Argentina	End July	IPK
*C. sativa* CAN58	D	Spain	End August	IPK
*C. sativa* cv. Finola	D	Finland	End June	Seed company^b^
*C. sativa* cv. Carmagnola	D	Italy	Beginning August	Seed company^c^
*C. sativa* cv. Carmagnola Selezionata (CS)	D	Italy	Beginning August	CRA-CIN^d^
*C. sativa* cv. Fibranova	D	Italy	Beginning August	CRA-CIN^d^
*C. sativa* cv. Fedora	M^**^	France	Middle July	Seed company^e^
*C. sativa* cv. Futura 75	M	France	Middle July	Seed company^e^
*C. sativa* cv. Felina 32	M	France	Middle July	Seed company^e^
*C. sativa* cv. Ferimon	M	France	Middle July	Seed company^e^
*C. sativa* cv. Codimono	M	Italy	Middle July	CRA-CIN^f^
*C. sativa* cv. Carmaleonte	M	Italy	Middle July	CRA-CIN^f^
*C. sativa* cv. Kc Dora	M	Hungary	End July	Seed company^g^

a *IPK, Leibniz Institute of Plant Genetics and Crop Plant Research, Gatersleben, Germany*.

b *Exchange with other scientists. Seed company: Finola Ltd, http://www.finola.fi/*.

c *Assocanapa srl, Carmagnola (Torino, Italy) http://www.assocanapa.org/*.

d *CRA-CIN, Centro di ricerca per le colture industriali, Rovigo (IT) from dr. G. Grassi*.

e *Cooperative Centrale des Producteurs de Semences de Chanvre (Le Mans, France)*.

f *CRA-CIN, Centro di ricerca per le colture industriali (CRA-CIN), Bologna (IT) from dr. A. Di Candilo*.

g *Agromag Kft, Hungary, http://www.agromag.hu/en*.

D

* , Dioecious plant; M

** *, Monoecious plant*.

### Total protein and antinutritional compound analysis

Samples of whole seeds were ground in a mortar and defatted by extracting with hexane (1:10, w/v). Protein was extracted from defatted flours using a Plant Total Protein Extraction Kit (Sigma-Aldrich, St. Louis, USA). The Plant Total Protein Extraction Kit includes two reagents, a plant specific protease inhibitor cocktail and a chaotropic reagent with increased solubilizing power to extract more hydrophobic proteins. Protein content was determined via a Quantum Protein Kit (Euroclone, Milan, Italy), using Bovine Serum Albumin (BSA) as standard.

Phytic acid was extracted from defatted flour as described by De Boland et al. (1975) with minor modifications. Briefly, the phytic acid was extracted with 0.4 N HCl plus 0.7 M Na_2_SO_4_ and then precipitated by 15 mM FeCl_3_ in 0.2 N HCl. The phosphorus content of the precipitate was colorimetrically determined, after acid digestion with sulfuric acid, according to Chen et al. (1956). Phytic acid content was determined by multiplying the phytate phosphorus content by a constant factor of 3.55 (Raboy and Dickinson, 1984).

Trypsin inhibitor (TI) was extracted from defatted flour with 2 mM glycine buffer pH 11 containing 2 mM NaCl, 10 mM urea, and 25 mM EDTA. Trypsin inhibitor activity was measured as described by Russo and Reggiani (2013). To 200 μL of extract were added 200 μL (40 μg.mL^−1^) of trypsin and then pre-incubated at 37°C for 3 min. After this, 500 μL (0.4 mg.mL^−1^) of N-benzoyl-DL-arginine-p-nitroanilide (pre-warmed to 37°C) were added to start the reaction. After incubation at 37°C for 10 min, 100 μL 30% (v/v) acetic acid was added to terminate the reaction and the mix centrifuged. Activity of trypsin was measured by the absorbance at 410 nm due to p-nitroaniline released. One unit of TI was defined as 0.01 decreases in absorbance at 410 nm under assay conditions compared with the control (without inhibitor).

### Total phenolic content, antioxidant activity and tocopherols

Phenols were extracted from defatted flours with 80% ethanol at 70°C (Russo and Reggiani, 2015c). The total phenolic (TP) content was determined using the Folin-Ciocalteu method according to Velioglu et al. (2006). One hundred microliters of the ethanolic extract or caffeic acid standard (50, 100, 200, 400 μg) was combined with 500 μL of Folin-Ciocalteu reagent and allowed to stand at room temperature for 5 min. Then, 400 μL of 60 g.L^−1^ sodium carbonate solution were added to the mix and the tubes were heated at 45°C for 15 min. The absorbance was measured at 765 nm after sitting for 30 min in the dark. The results were expressed as caffeic acid equivalents per g of dry weight (g CAE kg^−1^ DW).

The antioxidant activity of the ethanolic extracts was determined by the Antioxidant Assay Kit (Sigma-Aldrich, Milan, Italy) according to the manufacturer's protocol. In the kit, trolox (a water-soluble vitamin E analog) was present as a standard for antioxidant control. Trolox has been broadly applied in assaying food samples (Re et al., 1999; Huang et al., 2005; Russo and Reggiani, 2015c). The results were expressed as mol trolox equivalents per kg of dry weight (mol TE kg^−1^ DW).

Tocopherols were analyzed in HPLC by direct injection of oil (Gimeno et al., 2000). The tocopherol isomers were separated using an HPLC Jasco Tritotar III and Jasco MD910 Diode Array Detector (DAD). An amount of 5–10 μl of pure oil was loaded into a Kinetex 2.6 μm C18 100A (Phenomenex, Castel Maggiore, Italy) column (100 × 4.6 mm) and eluted with 1.5 ml.min^−1^ MeOH 95%. The DAD spectra gave information on the purity of tocopherols. Absorbance at 280 nm was elaborated by the Borwin software system to determine tocopherol amounts in comparison with standards (Sigma-Aldrich, St. Louis, USA).

### Oil content and fatty acid composition

Lipid extraction was performed according to Hara and Radin (1978). The fatty acid composition was determined in triplicate by gas chromatography (GC) of the oils after transesterification according to Christie (1982) with the modifications described by Chouinard et al. (1999). Gas chromatography analyses were carried out on an Agilent 6890 GC system (Paolo alto, CA) equipped with autosampler, on column injector, and a FID detector. The separation was performed on a 100% dimethylpolysiloxane column (CP-Sil 88 for FAME, 100 m × 0.25 mm × 25 μm) adopting the following conditions. The carrier gas was H_2_ at a constant pressure of 28 psi. Column temperature was set at 190°C for 22 min, and then increased to 240°C at a rate of 10°C min^−1^ and maintained for 13 min. Fatty acid methyl esters (FAMEs) were identified by comparison of their retention times with known standards (37-component FAME mix, Supelco 47885-U) and expressed as relative percentage of total fatty acids.

### Statistical analyses

All analyses were carried out in triplicate. Analysis of Variance (ANOVA) was applied to establish significant differences (*P* ≤ 0.01) between hemp genotypes in the levels of seed traits using SPSS version 16.0 software. Mean separation was performed using Duncan's test and referring to *P* ≤ 0.05 probability level. Pearson's correlations between seed traits were also calculated.

## Results

**Figure 1:**
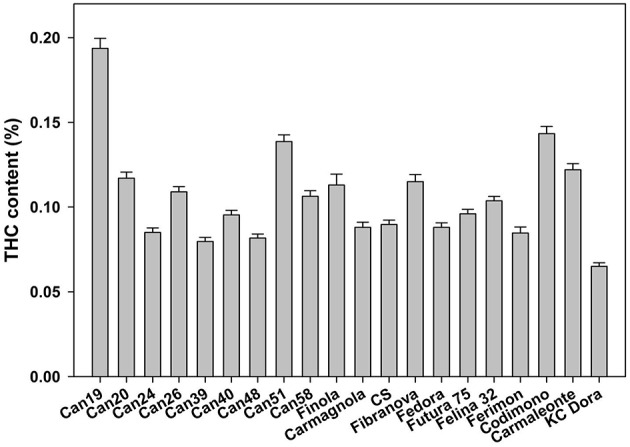
**THC content in inflorescences of hemp accessions (from CAN19 to CAN58) and cultivars (from cv. Finola to cv. Kc Dora)**.

All hemp plants considered in this study were analyzed for THC content (Figure 1). They showed THC concentrations between 0.07 and 0.19% in their inflorescences, which are below the authorized limit of 0.20% on a DM basis set in Italy.

### Protein and antinutritional compounds

Hemp seed meal was found to be a rich protein source with a mean protein content of 337 g.kg^−1^ of DW (Table 2). ANOVA analysis revealed significant differences in protein content among cvs and accessions. The CAN20 and CAN40 accessions showed the highest protein concentration in the meal (356 and 354 g.kg^−1^ DW, respectively). Pearson analysis demonstrated that the total protein content was highly correlated with TSW indicating that protein is an important component of seed weight (Table 3). A positive correlation is also evident among protein content and antioxidant activity (Table 3).

**Table 2:** **Seed traits in hemp accessions (from CAM19 to CAN58) and cultivars (from Finola to Kc Dora)**.

	**TSW^a^**	**Oil^b^**	**ALA(ω3)^c^**	**LA(ω6)^c^**	**Toc^d^**	**Protein^d^**	**Phytate^d^**	**TI^e^**	**TP^f^**	**Antiox^g^**
CAN19	17.5 (efgh)	32.5 (defg)	15.9 (n)	60.2 (b)	0.76 (gh)	345 (cd)	53.0 (f)	18.4 (f)	7.34 (gh)	1.66 (abc)
CAN20	19.1 (cd)	29.5 (hij)	18.5 (g)	59.4 (de)	0.82 (efgh)	356 (a)	60.1 (de)	13.8 (k)	9.01 (bc)	1.67 (abc)
CAN24	13.9 (i)	32.7 (cdef)	28.6 (a)	46.6 (m)	0.85 (defg)	331 (ef)	60.3 (de)	17.1 (g)	6.93 (h)	1.22 (g)
CAN26	21.7 (b)	34.9 (ab)	19.3 (f)	57.1 (g)	0.85 (defg)	345 (c)	44.6 (h)	15.8 (hij)	8.65 (cd)	1.63 (bcd)
CAN39	9.4 (k)	30.4 (ghij)	15.8 (o)	55.2 (j)	0.60 (i)	320 (gh)	53.7 (f)	11.3 (l)	7.90 (ef)	1.44 (f)
CAN40	19.3 (cd)	34.8 (abc)	20.5 (c)	54.0 (l)	0.69 (hi)	354 (ab)	43.8 (h)	16.3 (ghi)	7.83 (fg)	1.46 (f)
CAN48	22.8 (a)	34.0 (abcd)	12.8 (q)	58.7 (f)	0.75 (gh)	339 (cde)	73.3 (a)	18.9 (ef)	8.39 (de)	1.59 (de)
CAN51	21.6 (b)	35.4 (ab)	20.0 (e)	56.1 (i)	0.79 (fgh)	336 (de)	65.5 (b)	20.2 (cd)	7.86 (fg)	1.46 (f)
CAN58	16.7 (h)	36.0 (a)	20.2 (d)	54.4 (k)	0.86 (defg)	346 (bc)	61.7 (cd)	16.2 (ghij)	9.03 (bc)	1.66 (abc)
Finola	11.6 (j)	32.7 (cdef)	21.1 (b)	59.6 (cd)	0.90 (defg)	317 (gh)	63.0 (bcd)	16.2 (ghij)	5.88 (i)	0.92 (h)
Carmagnola	21.4 (b)	31.0 (fghi)	18.4 (g)	56.4 (h)	1.10 (bc)	334 (ef)	52.7 (f)	19.4 (de)	9.03 (bc)	1.62 (bcd)
CS	19.3 (cd)	31.6 (efgh)	17.8 (i)	59.7 (cd)	1.10 (b)	316 (h)	58.5 (e)	21.1 (c)	9.22 (b)	1.60 (de)
Fibranova	18.3 (de)	30.7 (fghij)	15.7 (o)	58.6 (f)	1.35 (a)	325 (fg)	53.1 (f)	10.8 (l)	9.40 (b)	1.62 (bcd)
Fedora	17.8 (efg)	29.2 (ij)	16.3 (l)	59.7 (c)	0.93 (def)	339 (cde)	64.4 (bc)	21.0 (c)	7.51 (fg)	1.56 (e)
Futura 75	17.9 (ef)	29.8 (hij)	17.5 (j)	60.1 (b)	0.96 (cde)	337 (cde)	75.0 (a)	27.8 (a)	10.29 (a)	1.68 (ab)
Felina 32	16.8 (gh)	29.6 (hij)	16.0 (n)	60.2 (b)	0.98 (bcd)	343 (cd)	75.5 (a)	26.6 (b)	10.63 (a)	1.70 (a)
Ferimon	17.1 (fgh)	30.1 (hij)	16.2 (m)	62.0 (a)	0.95 (de)	344 (cd)	72.7 (a)	26.4 (b)	10.11 (a)	1.68 (ab)
Codimono	17.3 (efgh)	33.8 (bcde)	18.2 (h)	56.4 (hi)	0.97 (bcde)	336 (de)	48.1 (g)	15.4 (ij)	9.09 (bc)	1.67 (abc)
Carmaleonte	19.3 (cd)	28.5 (j)	13.1 (p)	59.1 (e)	0.61 (i)	345 (cd)	44.3 (h)	15.2 (j)	10.11 (a)	1.67 (abc)
Kc Dora	19.5 (c)	31.5 (fgh)	16.8 (k)	56.2 (hi)	0.88 (defg)	332 (ef)	43.7 (h)	16.9 (gh)	9.00 (bc)	1.66 (abc)
Mean ± SEM	17.9 ± 0.4	31.9 ± 0.3	17.9 ± 0.4	57.5 ± 0.4	0.88 ± 0.04	337 ± 2	58.4 ± 1.4	18.2 ± 0.6	8.66 ± 0.16	1.56 ± 0.02
P genotype	< 0.01	< 0.01	< 0.01	< 0.01	< 0.01	< 0.01	< 0.01	< 0.01	< 0.01	< 0.01
P group^*^	ns	< 0.01	< 0.05	< 0.01	< 0.01	< 0.01	ns	< 0.01	< 0.01	ns

a *Data expressed as g.1000 seeds*.

b *Data expressed as % of seed weight*.

c *Data expressed as % of total fatty acids*.

d *Data expressed as g.kg^−1^ of oil*.

e *Data expressed as unit.mg^−1^*.

f *Data expressed as g CAE kg^−1^*.

g *Data expressed as mol TE kg^−1^*.

Means with different letters in parentheses within the same row differ significantly by Duncan's range test (P ≤ 0.05);

* *group cvs vs. group accessions. TSW, Thousand seed weight; Toc, tocopherols; TI, Trypsin inhibitor activity; TP, Total phenolic content; Antiox, Antioxidant activity*.

**Table 3:** **Pearson correlation coefficient (r) among seed traits in twenty hemp genotypes**.

	**TSW**	**Oil**	**ALA(ω3)**	**LA(ω6)**	**Toc**	**Protein**	**Phytate**	**TI**	**TP**
Oil	0.192	1							
ALA(ω3)	–0.280^*^	0.386^**^	1						
LA(ω6)	0.205	–0.424^**^	–0.749^**^	1					
Toc	0.132	–0.106	0.034	0.189	1				
Protein	0.402^**^	0.074	–0.080	0.065	–0.314^*^	1			
Phytate	–0.090	–0.168	–0.058	0.315^*^	0.150	–0.035	1		
TI	0.189	–0.174	–0.089	0.386^**^	0.163	0.127	0.679^**^	1	
TP	0.346^**^	–0.367^**^	–0.486^**^	0.389^**^	0.272^*^	0.259^*^	0.140	0.379^**^	1
Antiox	0.540^**^	–0.198	–0.580^**^	0.372^**^	0.152	0.463^**^	–0.035	0.237	0.775^**^

** Correlation is significant at the 0.01 level;

* *Correlation is significant at the 0.05 level*.

*TSW, Thousand seed weight; Toc, tocopherols; TI, Trypsin inhibitor activity; TP, Total phenolic content; Antiox, Antioxidant activity*.

Phytate content was very high in all tested meals (mean 5.84%). The lowest phytate content was detected in Kc Dora 4.37% and CAM40 4.38%, whereas cv Felina meal showed almost twice that amount (7.55%; Table 2). Regarding the antinutritional TI, Table 2 shows that its activity varied from 10.8 to 27.8 unit.mg^−1^ of defatted flour. Among hemp genotypes, Fibranova exhibited the lowest TI activity (Table 2). ANOVA analysis also evidenced significant differences in TI activity between cvs and accessions (cvs are on average richer in TI). Pearson analysis indicated that TI activity is highly correlated to phytate content (Table 3).

### Tocopherols, total phenolic content, and antioxidant activity

All hemp seeds analyzed in this study contained primarily γ-tocopherol and lesser quantities of α- and δ-tocopherols (data not shown). Table 2 reports the total content of tocopherols including all isomers identified. The average of tocopherol concentration for hemp genotypes was 0.88 g.Kg^−1^ of oil. ANOVA analysis showed that the differences in tocopherols among all genotypes and groups (cvs vs. accessions) are significant at 0.01 level. A similar result was observed for TP content (Table 2). The highest TP content was detected in some monoecious cultivars (see those with “a” letters in parenthesis by the Duncan's range test). According to Table 2, TP varied from 5.88 to 10.63 mg CAE g^−1^ DW of defatted flour. Pearson analysis showed that TP content was positively correlated to antioxidant activity (*r* = 0.775, Table 3) and tocopherols (*r* = 0.275) at the 0.01 and 0.05 levels, respectively. These data support the notion that phenols and tocopherols are effective scavengers of free radicals. As can be seen in Table 2, antioxidant activity ranged from 0.92 to 1.70 mol TE kg^−1^ DW of defatted flour. The cv Felina exhibited the highest antioxidant activity (Table 2).

### Oil content and composition

The oil content of the hemp seed genotypes ranged from 28.5 to 36.0% (Table 2) with an average of 31.9%. The highest oil content was measured in CAN58 (36%) and the lowest in Carmaleonte (28.5%). ANOVA revealed significant differences among genotypes for oil content at the 0.01 level.

The fatty acid composition of hemp seed oils is shown in Table 4. The principal saturated fatty acid was the palmitic acid (PA; 16:0) in all genotypes ranging between 5.98 and 8.60%, followed by stearic acid (SA; 18:0), varying from 2.26 to 4.61%. The saturated fatty acid fraction represents on average 10.8% of the total fatty acids (Table 5). The major monounsaturated fatty acid was oleic acid (OA; 18:1*cis*9, average of 13.4%) (Table 4) and CAN39 seeds showed the highest OA (16.8%) content. With respect to the essential fatty acids, the seeds of the analyzed genotypes contained on average 55.7% of LA and 17.4% of ALA, resulting in a ω6:ω3 ratio = 3.3:1 (Table 5). Significant differences among the genotypes analyzed were observed for LA (from 46.1 to 58.1%), the fatty acid present in hemp seed in the greatest proportion. Furthermore, noticeable differences in ALA concentration were observed, with the greatest amount measured in CAM24 (28.4%) and the lowest quantity in CAN48 and Carmaleonte (~12%).

**Table 4:** **Oil composition in twenty hemp seed accessions (from CAN19 to CAN58) and cultivars (from cv. Finola to cv. Kc Dora)**.

	**PA 16:0**	**PAL 16:1**	**SA 18:0**	**OA 18:1*cis*9**	**LA 18:2ω6**	**GLA 18:3ω6**	**ALA 18:3ω3**	**SDA 18:4ω3**	**ARA 20:0**	**GOA 20:1*cis*11**	**BA 22:0**
CAN19	6.05 (ij)	0.12 (j)	2.60 (h)	13.6 (g)	56.7 (c)	3.48 (c)	14.9 (m)	0.99 (bc)	0.86 (abc)	0.34 (abcd)	0.34 (ab)
CAN20	5.98 (j)	0.12 (j)	3.03 (ef)	11.5 (m)	57.9 (a)	1.46 (i)	18.0 (f)	0.51 (f)	0.90 (ab)	0.34 (abcd)	0.32 (abc)
CAN24	8.60 (a)	0.15 (def)	3.03 (ef)	11.5 (m)	46.1 (j)	0.49 (p)	28.4 (a)	0.23 (jk)	0.85 (abc)	0.37 (ab)	0.34 (ab)
CAN26	7.30 (b)	0.16 (cde)	2.62 (h)	12.3 (k)	56.1 (d)	0.94 (k)	18.9 (e)	0.34 (h)	0.67 (cdef)	0.31 (bcde)	0.27 (bcde)
CAN39	7.09 (bc)	0.16 (cde)	3.69 (b)	16.8 (a)	54.5 (g)	0.65 (n)	15.7 (j)	0.16 (l)	0.77 (bcde)	0.29 (cdef)	0.24 (bcde)
CAN40	6.79 (cde)	0.13 (hij)	3.34 (d)	13.9 (f)	51.8 (i)	2.27 (f)	19.8 (c)	0.75 (de)	0.71 (bcdef)	0.28 (def)	0.27 (bcde)
CAN48	7.35 (b)	0.19 (b)	2.91 (g)	16.3 (b)	57.1 (b)	1.54 (h)	12.4 (n)	0.32 (hi)	1.00 (a)	0.39 (a)	0.42 (a)
CAN51	5.98 (j)	0.12 (j)	2.95 (fg)	13.5 (g)	55.4 (f)	0.74 (m)	19.7 (c)	0.27 (ij)	0.75 (bcde)	0.31 (bcde)	0.30 (bcd)
CAN58	6.97 (cd)	0.12 (j)	4.61 (a)	12.5 (j)	53.7 (h)	0.62 (n)	19.9 (b)	0.24 (j)	0.73 (bcdef)	0.28 (def)	0.25 (bcde)
Finola	5.99 (j)	0.14 (fghi)	2.26 (i)	9.2 (n)	55.0 (f)	4.54 (a)	19.6 (d)	1.54 (a)	1.02 (a)	0.34 (abcd)	0.35 (ab)
Carmagnola	7.31 (b)	0.20 (b)	3.49 (c)	13.2 (h)	55.3 (f)	1.19 (j)	17.9 (f)	0.49 (f)	0.57 (ef)	0.23 (f)	0.15 (e)
CS	6.16 (hij)	0.15 (def)	2.32 (i)	12.7 (i)	58.2 (a)	1.46 (i)	17.3 (g)	0.49 (f)	0.60 (ef)	0.31 (bcde)	0.27 (bcde)
Fibranova	6.94 (cde)	0.14 (fghi)	2.87 (g)	14.4 (e)	57.4 (b)	1.21 (j)	15.3 (k)	0.42 (g)	0.77 (bcde)	0.33 (abcd)	0.30 (bcd)
Fedora	6.38 (gh)	0.17 (c)	2.97 (fg)	13.2 (h)	56.5 (c)	3.23 (d)	15.3 (k)	0.98 (c)	0.73 (bcdef)	0.36 (abc)	0.19 (de)
Futura 75	6.34 (ghi)	0.24 (a)	3.03 (ef)	11.9 (l)	58.0 (a)	2.14 (g)	16.8 (h)	0.71 (e)	0.52 (f)	0.25 (ef)	0.15 (e)
Felina 32	6.03 (ij)	0.16 (cd)	2.57 (h)	13.9 (f)	57.9 (a)	2.28 (f)	15.2 (kl)	0.77 (d)	0.62 (def)	0.32 (bcde)	0.20 (cde)
Ferimon	6.44 (fgh)	0.16 (cd)	2.53 (h)	11.4 (m)	58.1 (a)	3.85 (b)	15.1 (l)	1.04 (b)	0.74 (bcde)	0.30 (bcde)	0.29 (bcd)
Codimono	6.72 (def)	0.13 (ghij)	2.94 (fg)	14.3 (e)	55.8 (e)	0.57 (o)	18.0 (f)	0.18 (kl)	0.77 (bcde)	0.31 (bcde)	0.33 (ab)
Carmaleonte	7.34 (b)	0.15 (de)	3.13 (e)	15.7 (c)	56.7 (c)	2.43 (e)	12.6 (n)	0.55 (f)	0.88 (abc)	0.22 (f)	0.35 (ab)
Kc Dora	6.64 (efg)	0.14 (efgh)	3.45 (c)	15.4 (d)	55.3 (f)	0.90 (l)	16.5 (i)	0.26 (j)	0.82 (abcd)	0.30 (bcde)	0.32 (abc)
Mean ± SEM	6.72 ± 0.15	0.15 ± 0.01	3.02 ± 0.12	13.4 ± 0.43	55.7 ± 0.64	1.80 ± 0.27	17.4 ± 0.79	0.56 ± 0.08	0.76 ± 0.03	0.31 ± 0.01	0.28 ± 0.02
P genotype	< 0.01	< 0.01	< 0.01	< 0.01	< 0.01	< 0.01	< 0.01	< 0.01	< 0.01	< 0.01	< 0.01
P group^*^	ns	< 0.01	< 0.05	ns	< 0.01	< 0.01	< 0.01	< 0.01	ns	ns	ns

Data are expressed as % of oil fraction;

* *group cvs vs. group accessions. PA, palmitic acid; PAL, palmitoleic acid; SA, stearic acid; OA, oleic acid; LA, linoleic acid; GLA, γ-linolenic acid; ALA, α-linolenic acid; SDA, stearidonic acid; ARA, arachidic acid; GOA, gondoic acid; BA, behenic acid*.

*Means with different letters in parentheses within the same row differ significantly by Duncan's range test (P ≤ 0.05)*.

**Table 5:** **Saturated fatty acid (SFA), monounsaturated fatty acid (MUFA), polyunsaturated fatty acid (PUFA), and LA:ALA (ω6:ω3) ratio in twenty hemp accessions (from CAN19 to CAN58) and cultivars (from cv. Finola to cv. Kc Dora)**.

	**SFA**	**MUFA**	**PUFA**	**LA:ALA ratio**
CAN19	9.8	14.1	76.1	3.78
CAN20	10.2	11.9	77.8	3.22
CAN24	12.8	12.0	75.2	1.63
CAN26	10.9	12.8	76.4	2.96
CAN39	11.8	14.2	71.0	3.49
CAN40	11.1	14.3	74.6	2.63
CAN48	11.7	16.9	71.4	4.60
CAN51	10.0	13.9	76.1	2.80
CAN58	12.6	12.9	74.6	2.69
Finola	9.6	9.7	80.7	2.82
Carmagnola	11.5	13.6	74.9	3.06
CS	9.4	13.2	77.5	3.36
Fibranova	10.9	14.8	74.3	3.72
Fedora	10.3	13.7	76.0	3.67
Futura 75	10.0	12.4	77.6	3.44
Felina 32	9.4	14.4	76.2	3.77
Ferimon	10.0	11.9	78.1	3.84
Codimono	10.8	14.7	74.5	3.11
Carmaleonte	11.7	16.1	72.2	4.52
Kc Dora	11.2	15.9	72.9	3.35
Mean ± SEM	10.8 ± 0.2	13.7 ± 0.4	75.4 ± 0.5	3.32 ± 0.15

*Data are expressed as % of total fatty acids*.

The other fatty acids: palmitoleic (POA; 16:1), GLA, SDA, arachidic (ARA; 20:0), gondoic (GOA; 20:1*cis*11), and behenic acid (BA; 22:0) were found in small amounts. However, the percentage of GLA was the highest among the other minor unsaturated fatty acids and varied from 0.49 to 4.54%. The highest content of GLA was detected in Finola (4.54%) which also contained the highest quantity of SDA (1.54%), followed by Ferimon, CAN19 and Fedora with a content in GLA of 3.85, 3.48, 3.23%, respectively and of 1.04, 0.99, 0.98% of SDA, respectively (Table 4). ANOVA revealed significant variability among genotypes for all the fatty acids at the 0.01 level. Pearson correlation analysis showed that ALA is positively correlated with oil content and inversely correlated with LA, TP content and antioxidant activity (Table 3), whereas LA was positively correlated with antinutritional compounds (phytate and TI), TP content and antioxidant activity.

## Discussion

The possibility of using industrial hemp cvs with low levels of THC (< 0.20% THC on a DM basis as established by the European Union and also by Italian law) is leading to a re-introduction of this plant into the Italian production systems. In the 2015 about 2,000 hectares (ha) have been cultivated with hemp in Italy and in all countries of European Union its cultivation has reached 25,224 ha (European Industrial Hemp Association, http://eiha.org/downloads/). The interest on hemp is due to the huge number of products that can be obtained from this plant (Small and Marcus, 2002), and recently it is also largely focused on seeds, which are rich in healthy nutritional fats and proteins for both humans and animals (Mustafà et al., 1999; Rodriguez-Leyva and Pierce, 2010; Girgih et al., 2014).

In this work we have compared the seed composition of several cvs that are allowed to be cultivated in Europe with a collection of hemp accessions obtained from the IPK genebank and grown for 1 year in the same environment. Although hemp tend to perform better in its area of development (Dempsey, 1975), in our trial, except CAM58 which richest the flowering stage (see Table 1) and, consequently, seed maturation very late and cv Finola which was able to produce a discrete quantity of seeds but the plant remained very short, all the remaining hemp genotypes showed a good environmental adaptation.

In general, a high variability (*P* < 0.01) for all biochemical traits among the genotypes analyzed was observed (Table 2). Some of them, such as TSW, total protein, oil and ALA content appeared higher in hemp seed accessions than in cvs. In fact, protein content was >31% in all meals analyzed but CAN20 and CAN40 had the highest protein content ~35% and the latter also had an oil content of up to 34.8%.

As reported by others (Kriese et al., 2004; Chen et al., 2010; Vonapartis et al., 2015), the most abundant fatty acids in our hemp seed collection proved to be LA (average 55.7%), ALA (17.4%), and OA (13.4%), which together represent 86.5% of total fatty acids (Tables 4, 5). However, a high variability for LA and ALA content was detected among all hemp genotypes (Table 4) and the most different was CAN24 which showed 46.1 and 28.4% of LA and ALA respectively, giving a ratio ω6:ω3 (LA:ALA) of 1.63:1, which has never been reported before. In fact, a significant number of previous studies on hemp oil composition (Oomah et al., 2002; Callaway, 2004; Vonapartis et al., 2015) as well as most of the hemp genotypes analyzed in this study showed a ω6:ω3 ratio of roughly 3:1, reported as optimal for human nutrition (Erasmus, 1993; Leizer et al., 2000).

Simopoulos (2008) reported a ω6:ω3 ratio of 15–16.7:1 in Western European and American food supplies. This very high ratio, in today's diets, promotes the pathogenesis of many diseases, including cancer, cardiovascular and inflammatory and autoimmune diseases, whereas increased levels of ω3 polyunsaturated fatty acids (PUFAs) exert suppressive effects (Simopoulos, 2008; Gómez Candela et al., 2011). Therefore, the oil of CAN24 might be used, particularly in Western diets characterized by high LA and low ALA fatty acid intake, which is considered detrimental to health (Simopoulos, 2008; Gómez Candela et al., 2011).

In addition to LA and ALA, hemp seed oil also contains their direct metabolites GLA and SDA, which serve as intermediaries in the formation of longer-chain fatty acids and vital hormone-like prostaglandins in the body (Guil-Guerrero et al., 2010). The results of this study confirm that hemp seed oil is a good source of PUFA, especially GLA and SDA, but the content detected among the hemp seed genotypes varies greatly, even more than that detected for LA and ALA. As expected, the cv Finola showed the highest content of GLA (4.54%) and SDA (1.54%; Table 4) which is in agreement with results from other authors (Callaway, 2004; Vonapartis et al., 2015). However, an interesting level of GLA and SDA was also detected in CAN19, Fedora and Ferimon showing a content of GLA >3.0% and of SDA ~1%.

Hemp seeds showed a substantial content of tocopherols and total phenols (Table 2). In particular, TP content was higher in hemp seed than in flax, which is a species extremely rich in total phenols (Russo and Reggiani, 2015c). The high antioxidant activity detected in almost all hemp seeds, due to tocopherols and TP content, can ensure high oxidative stability that would makes hemp oil suitable for food and industrial applications, as well as improving the nutritional quality of human diets.

However, despite the functional and nutraceutical properties of this crop, the seeds are not totally free of antinutritional compounds, such as phytic acid, condensed tannins, cyanogenic glycosides, saponin and TI (Russo and Reggiani, 2013; Pojić et al., 2014). The quite high content (mean 5.84%) of phytate in our hemp collection confirmed the findings of Russo and Reggiani (2013) and the variability between the different hemp genotypes was statistically significant (Table 2). In some genotypes, the phytate content was more than 7% (those with letter “a” by Duncan's range test). Therefore, the use of such meal appears rather limited for monogastric animals and in human nutrition since a high level of phytic acid will affect protein digestibility, organoleptic properties, and bioavailability of macro- and microelements (Guillamón et al., 2008). Furthermore the high phytate content found, particularly in French hemp seed cultivars, will greatly limit the use of this protein source in novel food or feed formulations.

In addition, TI is responsible for the reduced digestibility of seed proteins and, for this reason, it constitutes one of the main antinutritional factor in plant seeds (Peric et al., 2013). As shown in Table 2, considerable variability in TI activity among all hemp meals was evident. However, the levels of TI in hemp seeds were lower in comparison with those observed in some cereals and soybean (Sosulki et al., 1988; Tsukamoto et al., 1995). Pearson coefficient (*r* = 0.679) indicated a strong positive correlation between TI activity and phytate content (Table 3) and this is in agreement with previous results reported by Russo and Reggiani (2015b) who analyzed six hemp cultivars grown in two different experimental fields for two consecutive years.

In conclusion our analysis confirmed that hemp seed is an excellent product in terms of protein content, oil composition and antioxidant molecules for food and feed formulations, but the content of some antinutritional compounds, particularly phytate will influence negatively its potential nutritional value. Therefore, an improvement for this trait would be necessary. Based on our data, some hemp accessions present in our germplasm collection, appear to contain more interesting traits than the cultivated hemp cvs. In fact, hemp accessions appeared more interesting then hemp cvs with regard to protein and oil content. In particular the Italian CAN40 showed a high content of both, therefore, might be used to improve oil and protein content in hemp cvs through hybridization and selection. Furthermore, the Italian accession CAM40 might be also a good candidate to reduce the content of phytate. In general, we observed that, among the genotypes analyzed, all Italian dioic and monoic cvs showed less phytate content than the monoic French cvs. This trend, except for CAN48, is also observed in the other Italian accessions. Therefore, our analysis evidenced that, although we analyzed a small segment of hemp germplasm collection and for only 1 year, probably an improvement of the cultivated hemp cvs for some traits might be possible using the large genetic variability present in the germplasm of hemp.

## Author contributions

This work was carried out in collaboration among authors. The authors of this manuscript worked together to design, conduct, analyze and interpret the findings of these experiments. All the authors read and approved the final manuscript.

### Conflict of interest statement

The authors declare that the research was conducted in the absence of any commercial or financial relationships that could be construed as a potential conflict of interest.
